# The disruption of mitochondrial axonal transport is an early event in neuroinflammation

**DOI:** 10.1186/s12974-015-0375-8

**Published:** 2015-08-28

**Authors:** Oihana Errea, Beatriz Moreno, Alba Gonzalez-Franquesa, Pablo M. Garcia-Roves, Pablo Villoslada

**Affiliations:** Center of Neuroimmunology, Institut d’Investigacions Biomèdiques August Pi Sunyer (IDIBAPS), Cellex Building, Laboratory 3A, Casanova 145, 08036 Barcelona, Spain; Diabetes and Obesity Research Laboratory, Institut d’Investigacions Biomèdiques August Pi Sunyer (IDIBAPS), 08036 Barcelona, Spain; Spanish Biomedical Research Center in Diabetes and associated disorders (CIBERDEM), University of Barcelona, 08907 Barcelona, Spain; Department of Physiological Sciences II, University of Barcelona, 08907 Barcelona, Spain; University of California, San Francisco, USA

**Keywords:** Mitochondria, Axonal transport, Axonal damage, Inflammation, Oxidative stress, Multiple sclerosis

## Abstract

**Background:**

In brain inflammatory diseases, axonal damage is one of the most critical steps in the cascade that leads to permanent disability. Thus, identifying the initial events triggered by inflammation or oxidative stress that provoke axonal damage is critical for the development of neuroprotective therapies. Energy depletion due to mitochondrial dysfunction has been postulated as an important step in the damage of axons. This prompted us to study the effects of acute inflammation and oxidative stress on the morphology, transport, and function of mitochondria in axons.

**Methods:**

Mouse cerebellar slice cultures were challenged with either lipopolysaccharide (LPS) or hydrogen peroxide (H_2_O_2_) ex vivo for 24 h. Axonal mitochondrial morphology was evaluated by transmission electron microscopy (TEM) and mitochondrial transportation by time-lapse imaging. In addition, mitochondrial function in the cerebellar slice cultures was analyzed through high-resolution respirometry assays and quantification of adenosine triphosphate (ATP) production.

**Results:**

Both conditions promoted an increase in the size and complexity of axonal mitochondria evident in electron microscopy images, suggesting a compensatory response. Such compensation was reflected at the tissue level as increased respiratory activity of complexes I and IV and as a transient increase in ATP production in response to acute inflammation. Notably, time-lapse microscopy indicated that mitochondrial transport (mean velocity) was severely impaired in axons, increasing the proportion of stationary mitochondria in axons after LPS challenge. Indeed, the two challenges used produced different effects: inflammation mostly reducing retrograde transport and oxidative stress slightly enhancing retrograde transportation.

**Conclusions:**

Neuroinflammation acutely impairs axonal mitochondrial transportation, which would promote an inappropriate delivery of energy throughout axons and, by this way, contribute to axonal damage. Thus, preserving axonal mitochondrial transport might represent a promising avenue to exploit as a therapeutic target for neuroprotection in brain inflammatory diseases like multiple sclerosis.

## Background

Axonal damage is the main factor contributing to the disability associated with many brain diseases caused by inflammation, oxidative stress, neurodegeneration, or ischemia. For example, in the case of an inflammatory disease like multiple sclerosis (MS), it is known that axonal damage occurs from the outset of the disease [1–3] and that it is the main contributor to long-term disability [4–6]. Axonal damage may be triggered by diverse mechanisms, progressing along the different phases of the disease. In the early inflammatory stages, axonal damage is mainly provoked by the inflammatory cascade, microglia activation, oxidative stress, and mitochondrial dysfunction [7–9]. By contrast, in more chronic stages, energy demands increase as a result of the loss of trophic support provided by myelin and the consequent redistribution of sodium channels, increasing the calcium inside axons and leading to axonal fragmentation due to the unsatisfied energy demands [10–12].

Disease progression in a mouse model of MS (experimental autoimmune encephalomyelitis (EAE)) is associated with reversible modifications to axons and other events that provoke axonal transection [13]. However, the timing of each event and the contribution of each process to transient and definitive axonal damage are not well understood. The identification of the early events leading to axonal damage after insult is critical to develop neuroprotective therapies that may decrease permanent axonal damage and disability in brain diseases such as MS.

Mitochondrial dysfunction seems to be one of the critical steps in axonal damage [14–16], and indeed, mitochondrial complex IV dysfunction has been detected in the axons, oligodendrocytes, and hypertrophied astrocytes of hypoxia-like pattern III MS lesions [17]. A microarray *postmortem* analysis of the non-myelinated motor cortex from MS patients revealed a reduction in the expression of 26 mitochondrial respiratory chain subunits and in the activity of complexes I, III, and IV [18]. Moreover, morphological changes to the mitochondria were detected inside axons undergoing focal axonal degeneration, as a step prior to the morphological changes and axonal fragmentation in EAE [13]. More recently, impaired axonal transport has been observed in EAE animals prior to the structural alterations to axons, their cargo, or microtubules. These deficits in transport began to revert within a few days of the peak of EAE, and they were reversed by anti-inflammatory and anti-oxidative interventions [19].

In this study, we set out to assess the contribution of mitochondrial abnormalities induced by inflammation or oxidative stress to axonal damage. By identifying the early processes involved in axonal damage, we should be able to promote the development of neuroprotective therapies targeting these processes. We performed in vivo imaging and molecular analyses of ex vivo murine cerebellar slice cultures challenged by agents that provoke inflammation (lipopolysaccharide (LPS)) or oxidative stress (H_2_O_2_). We focused on these two types of insult because they are particularly relevant to the acute inflammatory damage in MS relapses and, to some extent, they contribute to other diseases like brain trauma, ischemia, or neurodegeneration. Our results point to the disruption of mitochondrial axonal transport as an early pathogenic mechanism that triggers axonal damage during acute neuroinflammation.

## Materials and methods

### Cerebellar slice cultures and stimulations

Organotypic slice cultures were prepared from the cerebellum of 7-day-old C57BL/6J mice (Janvier Labs), obtaining 300 μm sagittal slices with a McIlwain Tissue Chopper (Mickle Laboratory). Three or four slices were placed on Cell Culture Inserts (Millipore) in six-well plates and cultured at 37 °C in an atmosphere of 5 % CO_2_ with 1 ml of organotypic slice medium: 50 % basal medium containing Earle’s salt (Gibco, Invitrogen), 25 % Hank’s buffered salt solution (Gibco, Invitrogen), 25 % inactivated horse serum (Gibco, Invitrogen), 5 mg/ml glucose (Sigma-Aldrich), 0.25 mM l-glutamine (Gibco, Invitrogen), 25 μg/ml penicillin/streptomycin (Gibco, Invitrogen), and 1× anti-mycotic (Gibco, Invitrogen). The medium was replaced every 2–3 days, and to reduce microglial activation and to permit axonal myelination, the slices were maintained for 7 days in vitro (DIV) before adding any stimulus. After 7 DIV, the slices were treated with *Escherichia coli* LPS (15 μg/ml; Sigma-Aldrich) or H_2_O_2_ (500 μM; Sigma-Aldrich) for different times (3, 6, 24, 48, 72, and 96 h) as described elsewhere [20]. Time course studies revealed that the earliest and most significant differences were observed 24 h after the challenge, and as such, this time point was used thereafter. The dose of H_2_O_2_ used previously for 24 h stimulations (1 mM H_2_O_2_) [20] was reduced to 500 μM to ensure better axonal survival at longer times of stimulation (data not shown). Untreated control slices were incubated for identical periods as the treated cultures: up to 8 DIV for 24 h stimulations and up to 11 DIV for experiments at different time points.

All animal handling was carried out in accordance with the European Council Directive (2010/63/EU) and the Spanish regulations for the procurement and care of experimental animals (1201 RD/2005, October 10). All the study protocols were approved by the Ethical Committee on Animal Research of the University of Barcelona.

### Transmission electron microscopy (TEM)

**Fig. 1:**
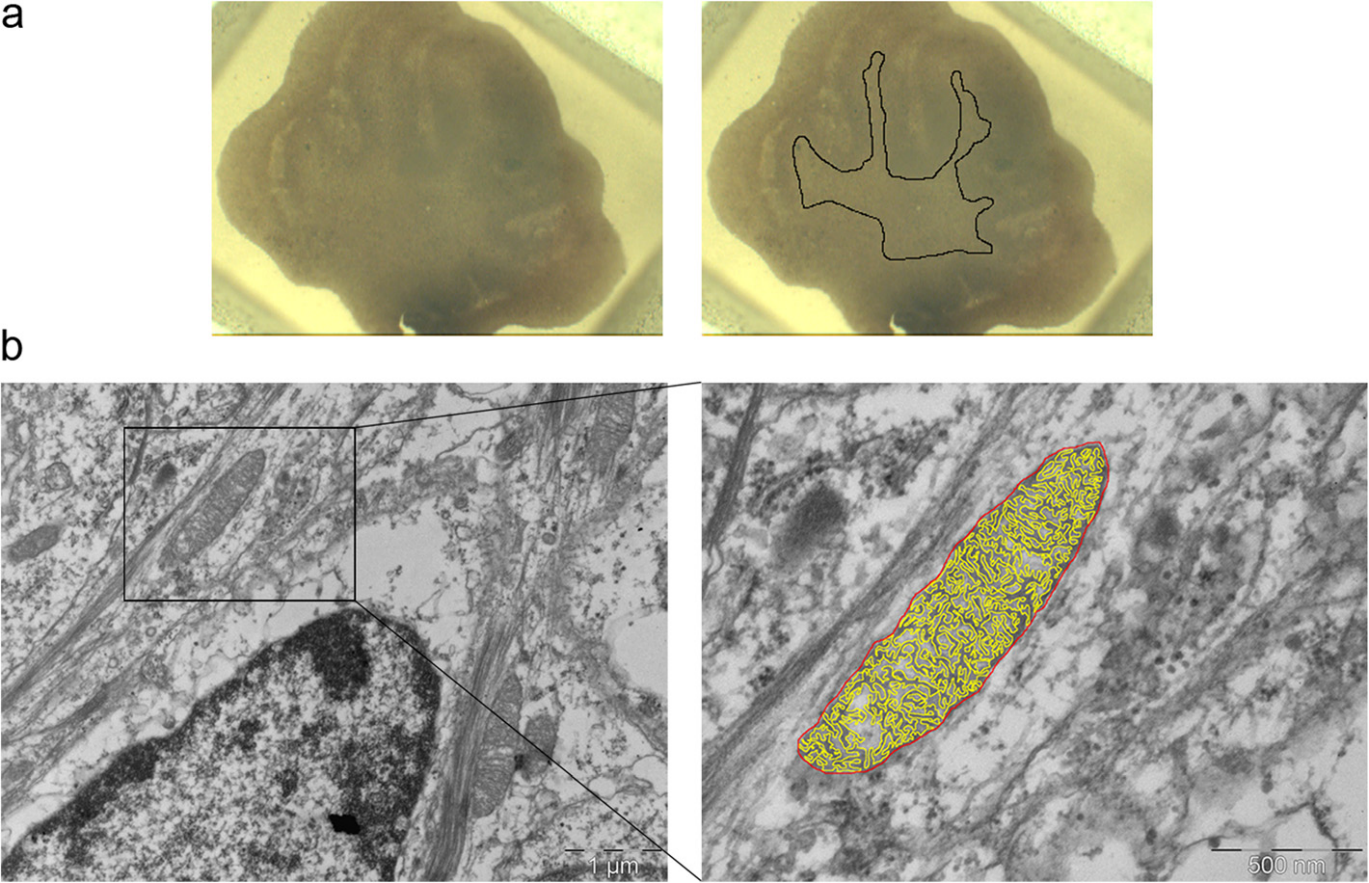
Analysis of axonal mitochondrial morphology by transmission electron microscopy in cerebellar slice cultures. **a** Images of an organotypic cerebellar slice culture embedded in an Epoxy resin block (*left* image) and the white matter area selected for ultramicrotomy (*area inside the black line*, *right* image). **b** Segmentation method to study axonal mitochondrial morphology. *Left* image shows a section of cerebellar white matter with a longitudinally cut axon and a mitochondrion inside. The external mitochondrial perimeter (*red line*, *right* image) and the internal cristae perimeter (*yellow line*, *right* image) were drawn manually using ImageJ. Scale bars, 1 μm left image and 500 nm right image

Cerebellar slices were fixed in 2 % paraformaldehyde and 2.5 % glutaraldehyde in 0.1 M phosphate buffer (PB) for 24 h at 4 °C, and after washing for 12 h with several changes of 0.1 M PB, the slices were post-fixed with 2 % osmium tetroxide in 0.1 M PB for 1 h at 4 °C. The tissue was dehydrated in increasing concentrations of methanol and flat embedded in Epoxy-embedding medium. Longitudinal sections were obtained along the original sagittal plane of the organotypic cultures, and only white matter areas were selected for ultramicrotomy (Fig. 1a). Ultra-thin sections were stained with a solution of 1 % uranyl acetate and lead citrate (all reagents from Sigma-Aldrich), and the sections were observed on a JEOL 1010 transmission electron microscope (JEOL, USA). Images of axonal mitochondria were acquired with a MegaView III digital camera (Olympus) and analyzed with ImageJ Software. Two different regions of interest were selected by hand for the analysis, the external mitochondrial perimeter, and the internal cristae perimeter (Fig. 1b). The mitochondrial morphology parameters are described in Table 1 [21, 22].

**Table 1:** Morphological parameters of mitochondria analyzed by transmission electron microscopy

Parameter	Meaning
Mitochondrial area	Total area of each mitochondrion
External perimeter	Mitochondrial external perimeter
Feret’s diameter	Longest distance between any two points of the mitochondrial external perimeter
Circularity	Two-dimensional sphericity index. A value of 1 corresponds to a perfect sphere. $\mathrm{Circ}=\frac{4\uppi \times \kern0.37em \mathrm{Area}}{{\left(\mathrm{Perimeter}\right)}^2}$
Roundness	Two-dimensional sphericity index. A value of 1 corresponds to a perfect sphere. $\mathrm{Round}=\frac{4\kern0.37em \times \kern0.37em \mathrm{Area}}{\uppi {\left(\mathrm{minoraxis}\right)}^2}$
Aspect ratio (AR)	Ratio between the major and the minor axis of a mitochondrion. $\mathrm{A}\mathrm{R}=\frac{\mathrm{Major}\ \mathrm{Axis}}{\mathrm{Minor}\ \mathrm{Axis}}$
Cristae perimeter/external perimeter	Ratio between the mitochondrial cristae perimeter and the external perimeter. Reflects the complexity of mitochondrial cristae

Description of the morphological mitochondrial parameters analyzed by TEM [21, 22]

### Preparation of lentiviral particles, microinjection into cerebellar slice cultures, and time-lapse imaging

A lentivirus containing mitochondrial-targeted DsRed2 was generously donated by Dr. Bruce D. Trapp (Department of Neurosciences, Cleveland Clinic Lerner Research Institute) [23]. The plasmid was amplified in competent DH5α *E. coli* (Invitrogen) transformed following the manufacturer’s instructions, and 293T cells were transfected with the construct and packaging plasmids using Lipofectamine 2000 (Invitrogen) according to the manufacturer’s protocol. Viral supernatants were collected 48 h after the transfection, centrifuged at 3000 *g* for 15 min and filtered through 0.45 μm polyvinylidene difluoride (PVDF) filters (Millipore) to eliminate the cell debris. Subsequently, the viral supernatants were centrifuged at 110,000 *g* for 3 h at 4 °C to concentrate the viral particles, which were resuspended in a 1/100 volume of PBS, aliquoted, and frozen at −80 °C until use.

Two hours after plating the cerebellar slices, the lentiviral particles were injected into the Purkinje cell layer using a microinjector (InjectMan, Eppendorf) and a micromanipulator (FemtoJet, Eppendorf) connected to a spinning disk confocal microscope (Revolution XDi System, Andor Technology) following the protocol described elsewhere [24]. Borosilicate capillaries with an external diameter of 2 mm (Warner Instruments) and stretched to a 5–15 μm diameter at the tip were used to perform three to four injections per slice.

**Fig. 2:**
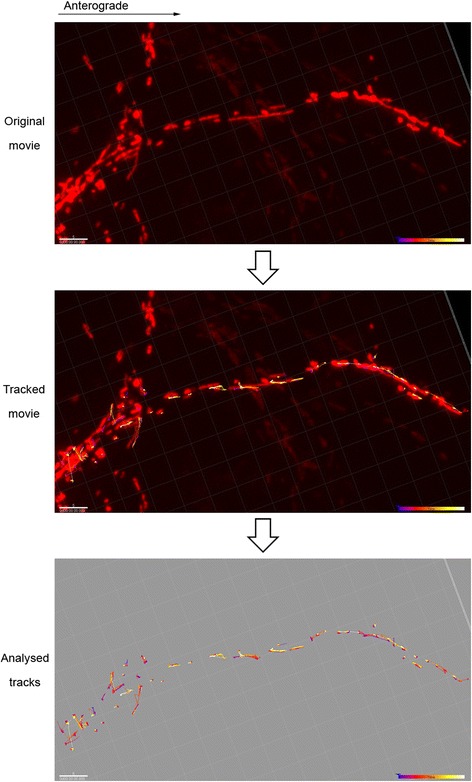
In vivo imaging of axonal mitochondrial transport in cerebellar slice cultures. (*Top*) Image stack extracted from an original recording showing an individual axon containing DsRed2-positive mitochondria. (*Middle*) Motile mitochondria were identified and tracked automatically using the autoregressive motion algorithm in the ImarisTrack package (Imaris software, Bitplane). (*Bottom*) The resulting tracks are time-color coded, and the software automatically calculates several parameters for each track, such as track area mean (μm^2^) or track speed mean (μm/s). Scale bars 5 μm, time bars 20 min

After 7 DIV and the corresponding stimulation, Purkinje cell axons with DsRed2-positive mitochondria were visualized as reported previously [23, 25]. Cell culture inserts with the slices attached were transferred to glass-bottom dishes (MatTek Corporation) with 1 ml of culture medium. Images were acquired for 20 min at 512 × 512 pixel resolution on an inverted spinning disk confocal microscope equipped with a cage incubator and an EMCCD camera (Revolution XDi System, Andor Technology). The resultant recordings were analyzed using Imaris software (Bitplane) that automatically generates tracks corresponding to moving objects (Fig. 2). Stationary mitochondria were identified as objects without any displacement during the recording time.

### High-resolution respirometry

High-resolution respirometry assays were performed on an Oxygraph-2k system (Oroboros Instruments, Austria), and for each measurement, 15 cerebellar slices were homogenized in 150 μl of MIR05 mitochondrial respiration buffer at pH 7.1 (0.5 mM EGTA, 3 mM MgCl_2_, 60 mM K-lactobionate, 20 mM taurine, 10 mM KH_2_PO_4_, 20 mM HEPES, 110 mM sucrose, 1 g/l BSA) [26]. Homogenized tissue (100 μl) was added to 2 ml of MIR05 in the different chambers of the respirometer. DatLab (Oxygraph-2k-associated software) provides respiratory values for the previously defined states by calculating the negative derivative of the oxygen concentration with respect to time.

The SUIT (substrate-uncoupler-inhibitor-titration) protocol used was as follows (the concentrations indicated are saturating and the final concentrations in the chambers): malate (2 mM; Sigma) and glutamate (10 mM; Sigma) to measure the LEAK uncoupled respiration state (due to proton leakage and the circuit of electrons and cations that is not dependent on ATP synthase activity); ADP (5 mM; Calbiochem) + MgCl_2_ (0.6 mols per each ADP mol) for the measurement of reduced nicotinamide adenine dinucleotide (NADH)-dependent respiration through complex I; the OXPHOS state, where the gradient of protons pumped into the mitochondrial intermembrane space is partially utilized by ATP synthase for oxidative phosphorylation, coupled respiration, and that is also partially dissipated due to proton leakage or uncoupled respiration; cytochrome C (10 μM; Sigma) was added to the chambers to verify if the mitochondrial membrane had been damaged during sample preparation (no significant increase in oxygen consumption was observed); succinate (10 mM; Sigma) to measure reduced flavin adenine dinucleotide (FADH_2_)-dependent complex II respiration together with complex I respiration in the OXPHOS CI + CII state; oligomycin (2 μg/ml; Sigma) to detect endogenous uncoupled respiration from ATP synthesis through complexes I and II substrates by inhibiting ATP synthase; carbonyl cyanide-*p*-trifluoromethoxyphenylhydrazone (FCCP) (0.5 + 0.5 μM; Sigma) until reaching the maximum respiration to quantify the maximum capacity of the electron transport system by non-physiological uncoupling of the internal mitochondrial membrane (ETS CI + CII state); rotenone (0.5 μM, Sigma) to inhibit complex I and thereby study the maximum respiration corresponding only to complex II (ETS CII); and antimycin A (2.5 μM; Sigma) to inhibit complex III and define the ROX (residual oxygen consumption) state that is related to non-mitochondrial respiration, in order to subtract this value from the previous values. Finally, ascorbate (2 mM; Sigma) and *N*,*N*,*N*′,*N*′-tetramethyl-*p*-phenylenediamine dihydrochloride (TMPD) (0.5 mM; Sigma) were added to analyze complex IV respiration. Ascorbate avoids TMPD self-oxidation and maintains it in its reduced state. Throughout the SUIT protocol, each reagent was added when the previous respiratory signal was stable for at least 2–3 min. After respirometry assays, the total protein in each sample was measured by the Bradford method (Sigma) to normalize the respiration values.

### Western blots

Four cerebellar slices were plated in each well, and after 7 DIV and the corresponding stimulations, the slices were collected in 100 μl of RIPA buffer (Sigma) supplemented with protease inhibitor cocktail (Sigma). The slices were homogenized, centrifuged for 5 min at 12,000 rpm and 4 °C, and the supernatant was recovered and the total protein was measured by the Bradford method (Sigma).

To detect SUO (70 kDa Fp subunit of the succinate ubiquinone oxidoreductase or complex II) and COX IV (subunit IV of the cytochrome c oxidase or complex IV), 40 μg of total protein was resolved on 4–12 % Criterion XT Bis-Tris gels (Bio-Rad) in MES buffer (Bio-Rad), more appropriate to separate low molecular weight proteins. Proteins were transferred to PVDF membranes (GE Healthcare), previously activated for 1 min with 100 % methanol. The membranes were then blocked for 1 h at room temperature with TBS (0.05 % Tween, 5 % dry milk) and incubated overnight at 4 °C with the primary antibodies diluted in blocking solution: mouse anti-SUO, 1:1000 (Molecular Probes) or mouse anti-COX IV, 1:1000 (Molecular Probes). After several washes with TBS-Tween 0.05 %, the membranes were incubated for 1 h at room temperature with the secondary horseradish peroxidase (HRP)-conjugated anti-mouse IgG antibody (GE Healthcare) diluted in blocking solution (1:10,000). Protein loading was assessed and normalized to the signal from a HRP-conjugated anti-β-actin antibody (Sigma) diluted 1:25,000 in blocking solution and incubated for 20 min at room temperature.

### ATP measurements

ATP production in the organotypic cerebellar slice cultures was measured using a bioluminescent assay (ATP Determination Kit, Molecular Probes) based on the ATP requirements of a recombinant firefly luciferase to produce light (emission maximum ~560 nm at pH 7.8). The experiments were carried out according to the manufacturer’s instructions. Briefly, the slices were collected from each well in 50 μl of deionized water and homogenized. Samples were transferred to a dark 96-well plate (Nunc), and a standard curve of different ATP concentrations was established from 0.001 to 1 nM. Finally, the plate was read in a luminometer (GloMax, Promega) using the following parameters: delay time 1 s, integration time 10 s, and injected reaction volume 100 μl.

### Statistical analysis

All statistical analyses were performed using SPSS 15.0 (IBM) and GraphPad Prism 6 (GraphPad Software) software, presenting the data as the mean ± SEM. The negative control and the LPS and H_2_O_2_ (24 h treatment) data were analyzed by one-way ANOVA and with a Dunnett’s multiple comparisons *post hoc* test when a Gaussian distribution was followed, or with a Kruskal-Wallis test and Dunn’s multiple comparisons *post hoc* test or a Mann-Whitney *U* test plus Bonferroni’s correction for *post hoc* multiple comparisons when samples didn’t follow a Gaussian distribution. For the experiments in which treatments were added at different time points, the data were analyzed by two-way ANOVA applying a Dunnett’s multiple comparisons *post hoc* test. Frequency analysis was performed with the chi-squared test. *P* values are presented as *p < 0.05, **p < 0.01, and ***p < 0.001.

## Results

### Inflammation and oxidative stress provoke an increase in the size and cristae complexity of axonal mitochondria

Changes to the mitochondria in axons undergoing inflammatory degeneration have previously been demonstrated in an animal model of MS and EAE [13]. Hence, we set out to characterize these morphological changes in axonal mitochondria in response to neuroinflammation (triggered by LPS-induced microglia activation) and oxidative stress (triggered by H_2_O_2_), in an ex vivo model using cerebellar slice cultures [20]. In such model, we have found that LPS also induces oxidative stress by inducing iNOS expression and increasing levels of reactive oxygen species [20]. Accordingly, longitudinally cut axons were identified in the white matter of cerebellar slices and the morphological parameters of the axonal mitochondria were analyzed in TEM images 24 h after the insult (Fig. 1 and Table 1).

**Fig. 3:**
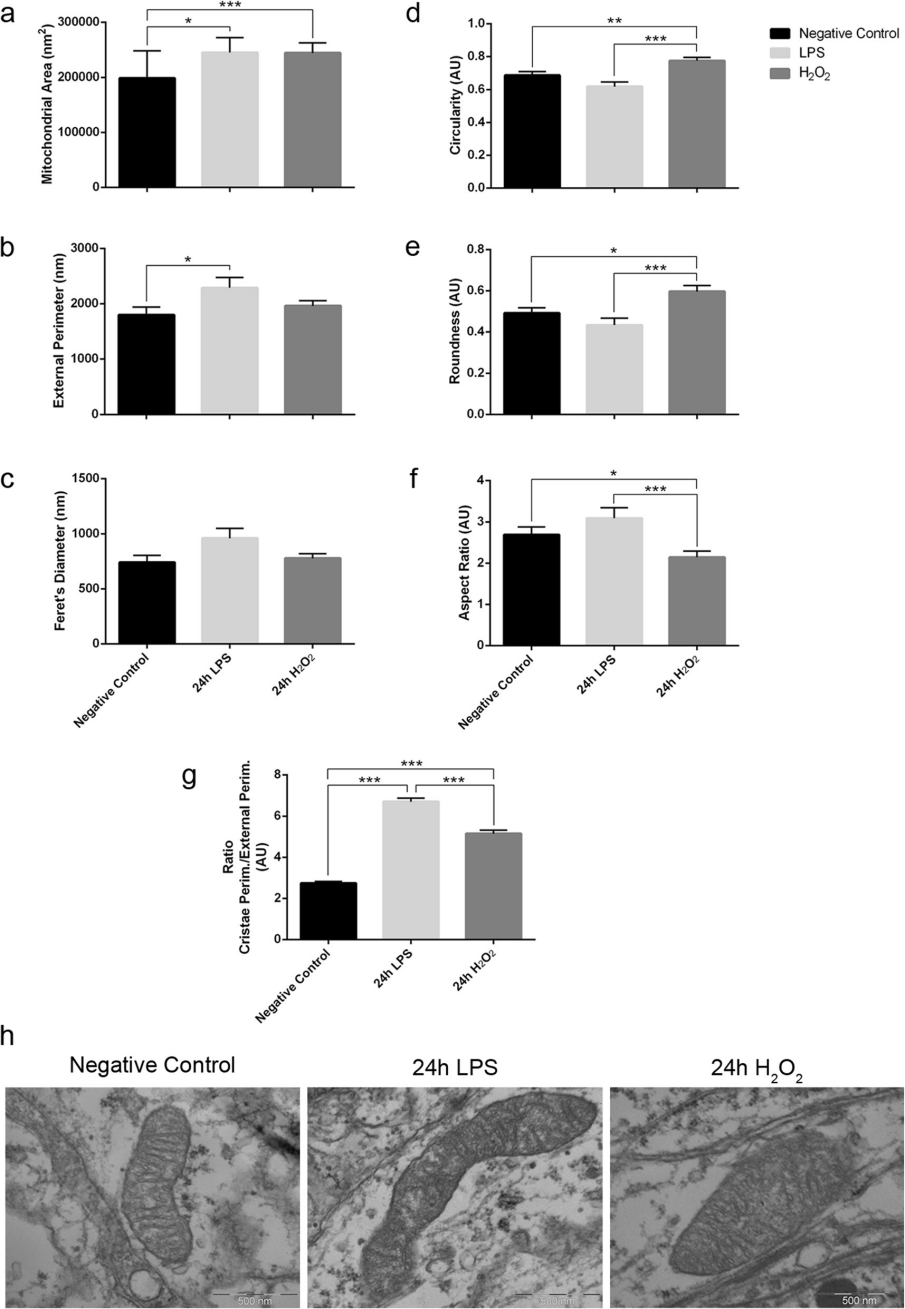
Effects of neuroinflammation and oxidative stress on axonal mitochondrial morphology. **a**-**c** Mitochondrial size parameters: **a** area, **b** external perimeter, and **c** Feret’s diameter. **a** Axonal mitochondria increase their area in conditions of neuroinflammation and oxidative stress. **b** However, the external perimeter only increases significantly during neuroinflammation, **c** while Feret’s diameter also has a tendency to increase in the neuroinflammatory condition. **d**-**f** Mitochondrial shape descriptors: **d** circularity, **e** roundness, and **f** aspect ratio. Axonal mitochondria are more rounded following oxidative stress than in unstimulated and LPS-stimulated cultures. **g** The ratio between the internal cristae perimeter and the external perimeter indicates that both neuroinflammation and oxidative stress induce an increase in axonal mitochondrial cristae complexity. **a**-**g** *n* = 73 control mitochondria, 50 LPS-challenged mitochondria and 75 H_2_O_2_-challenged mitochondria from two different experiments: Kruskal-Wallis test and Dunn’s multiple comparisons *post hoc* test. Mean ± SEM. (*AU* arbitrary units); *p < 0.05, **p < 0.01, ***p < 0.001. **h** Representative TEM images of axonal mitochondria from unstimulated, LPS-, and H_2_O_2_-stimulated organotypic cerebellar slice cultures. Scale bars 500 nm

Both these challenges increased the mitochondrial area compared to the untreated control (LPS vs control: *p* = 0.029; H_2_O_2_ vs. control: *p* = 0.001; Fig. 3a). Moreover, the external perimeter of the mitochondria increased following the neuroinflammatory challenge (*p* = 0.037 compared to the control) but not oxidative stress (*p* = 0.135; Fig. 3b). No differences were observed when considering Feret’s diameter (*p* = 0.069, Kruskal-Wallis test; Fig. 3c).

The shape of axonal mitochondria was analyzed using three different descriptors: circularity, roundness, and aspect ratio (Table 1). We did not find significant changes between the mitochondria in control and LPS-stimulated slices (Fig. 3d-f), although the axonal mitochondria in the slices exposed to LPS had a tendency to elongate (negative control, AR = 2.691 ± 0.191; LPS, AR = 3.092 ± 0.249; Fig. 3f). By contrast, axonal mitochondria were more rounded following oxidative stress than in the control slices (circularity *p* = 0.004, roundness *p* = 0.033, AR *p* = 0.033; Fig. 3-f).

Finally, as changes to the organization of the mitochondrial internal membrane regulate mitochondrial respiratory function [27, 28], the complexity of axonal mitochondrial cristae was analyzed, reflected by the ratio of the mitochondrial cristae perimeter and the external perimeter. This ratio increased beyond that of the controls after both challenges (LPS vs. control: *p* < 0.0001; and H_2_O_2_ vs. control: *p* < 0.0001; Fig. 3g, h). Thus, the acute response of axonal mitochondria to the inflammatory and oxidative insults in vitro involves an increase in their size and cristae complexity, suggesting compensatory changes in respiratory function.

### Changes in the distribution of stationary and motile axonal mitochondria after damage

Mitochondrial transport in axons is extremely important to correctly locate the mitochondria at sites of higher energetic demand, such as the nodes of Ranvier and synaptic terminals, as well as for mitochondrial renewal when the mitochondria that have lost their respiratory efficacy are destroyed [29–31]. Altered mitochondrial transport may compromise axonal survival even though mitochondrial function per se might not be disrupted. Therefore, we analyzed the acute changes in mitochondrial transport along axons after the inflammatory challenge and oxidative stress by in vivo imaging of the cerebellar slices in culture. Neuronal mitochondria were labeled by microinjection of lentiviral particles containing a mitochondrial-targeted DsRed2 sequence close to Purkinje cell somas, a lentivirus that preferentially infects neurons and not glial cells [23]. Purkinje cell axons with positive DsRed2 mitochondria were visualized 24 h after LPS or H_2_O_2_ challenge.

**Fig. 4:**
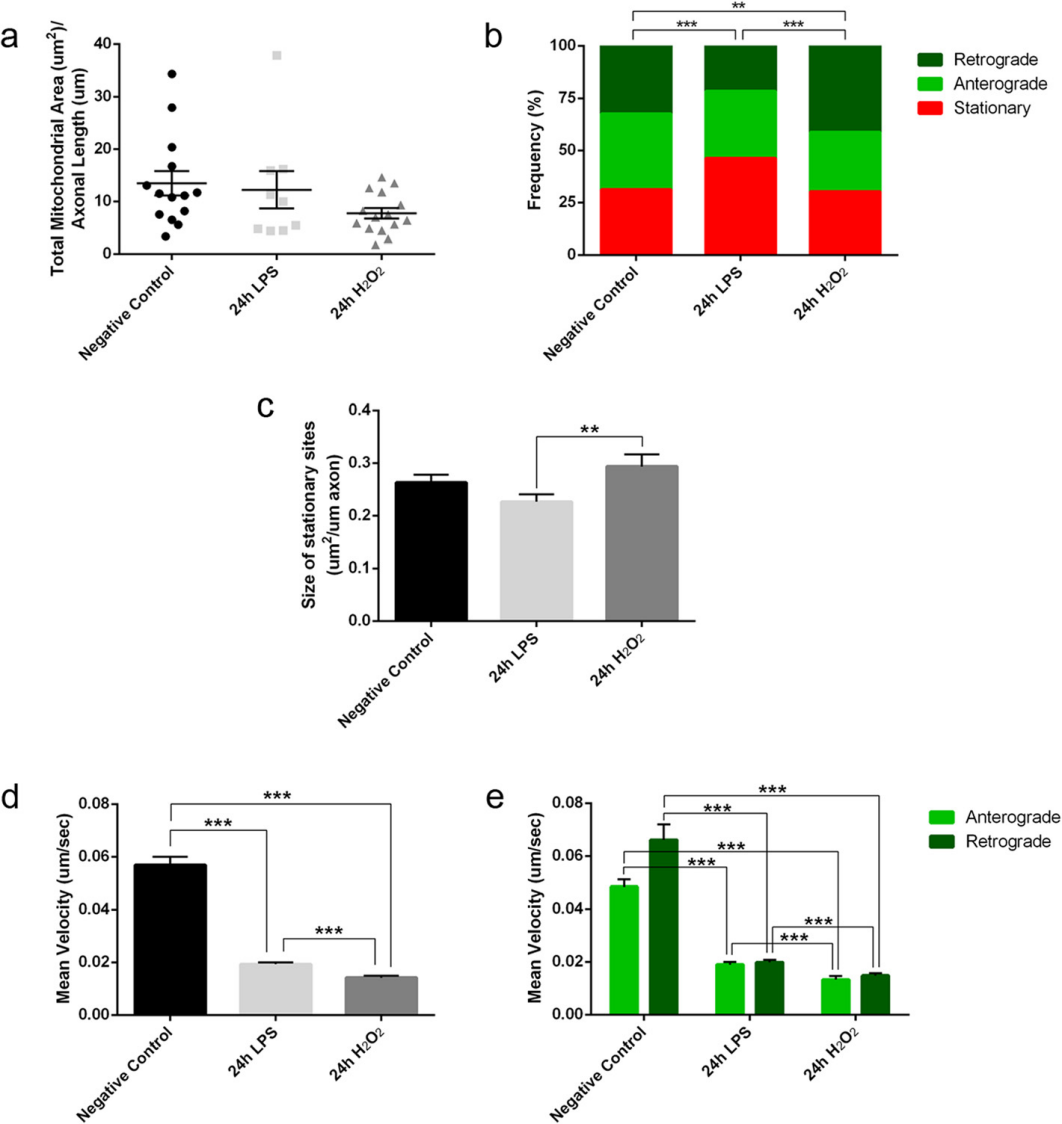
Inflammation and oxidative stress induce changes in axonal mitochondrial transport. **a** Axonal mitochondrial content does not vary after neuroinflammation or oxidative stress, although there is a slight tendency for oxidative stress to diminish mitochondrial density (Kruskal-Wallis test). **b** Frequency analysis of the distribution of motile and stationary mitochondrial populations shows that neuroinflammation produces an increase in stationary mitochondria and a decrease in retrograde motile mitochondria, whereas oxidative stress induces a significant increase in retrograde motile mitochondria (chi-squared test). **c** Neither challenge altered the size of the stationary sites with respect to the controls, yet there is a difference between both stimuli as smaller mitochondrial stationary sites are associated with neuroinflammation than with oxidative stress (Kruskal-Wallis test and Dunn’s multiple comparisons *post hoc* test). **d**, **e** Both stimuli dramatically decrease the mean velocity of motile mitochondria compared to the controls, even when the population of motile mitochondria is considered in terms of the orientation of the movement (Kruskal-Wallis test and Mann Whitney *U* test plus Bonferroni’s correction for *post hoc* multiple comparisons). **a**-**e** Three different experiments were performed where *n* = 614 control mitochondria, 444 LPS-challenged mitochondria, and 398 H_2_O_2_-challenged mitochondria, from 14 control axons, 9 LPS-challenged axons, and 15 H_2_O_2_-challenged axons: mean ± SEM. **p < 0.01, ***p < 0.001

We first quantified the total mitochondrial area in each axon registered and normalized this to the axon length to assess whether the total axonal mitochondrial content was affected by either challenge (LPS or H_2_O_2_). Axonal mitochondrial density was not affected by 24 h of neuroinflammation or oxidative stress (*p* = 0.198, Kruskal-Wallis test; Fig. 4a), indicating that the mitochondria were not significantly depleted from axons due to the damage induced.

Two different populations of the mitochondria can be distinguished in axons in terms of their transport: stationary mitochondria and motile mitochondria [32, 33]. In healthy axons, stationary mitochondria are located at the nodes of Ranvier where there is high energy demand required for the transmission of nerve impulses. Motile mitochondria move in an anterograde (towards the distal axon) or retrograde direction (towards the soma) from one site of stationary mitochondria to the next. Thus, the distribution of the different mitochondrial populations was analyzed in the three experimental conditions (Fig. 4b). In the control tissue, 31.3 % of the mitochondria were stationary and 68.7 % were motile (36.1 % anterograde and 32.6 % retrograde), whereas neuroinflammation increased the amount of stationary mitochondria to 46.2 %, decreasing the amount of retrograde motile mitochondria to 21.6 % (32.2 % anterograde; *p* < 0.0001 with respect to the controls, chi-squared test). By contrast, oxidative stress did not affect the percentage of stationary mitochondria (30.2 %), although the percentage of retrograde motile mitochondria increased significantly to 41.2 % (28.6 % anterograde; *p* = 0.010 with respect to the controls, chi-squared test).

### Axonal mitochondrial transport is severely disrupted by neuroinflammation and oxidative stress

Stationary mitochondria were identified as objects that were not displaced during the recording, although it is more accurate to name these as stationary mitochondrial sites since the resolution of axon recording did not allow us to distinguish if the fluorescent signal came from one or more mitochondria. The area occupied by objects that were not displaced within the stationary site was calculated and normalized to the axon’s length. There was a tendency for the size of the stationary sites to diminish following neuroinflammation but not oxidative stress (LPS vs control, *p* = 0.088; H_2_O_2_ vs. control, *p* = 0.719; Fig. 4c). Moreover, there was a significant difference in the size of stationary sites when the effect of the two stimuli was compared (*p* = 0.0059).

Motile mitochondria were identified as objects with a displacement velocity greater than zero at any moment in the recordings. Axonal mitochondria described diverse trajectories: maintaining the same direction, switching from anterograde to retrograde displacement and vice versa, moving at the beginning and then stopping, etc. For the mitochondria that go through a stationary site, it is impossible to know whether the same mitochondrion enters and leaves. Thus, in such cases, the trajectories were separated as belonging to different mitochondria. The parameter selected to assess the motile behavior of axonal mitochondria was the mean velocity [34], which decreased dramatically with respect to the controls after each challenge (LPS vs. control, *p* < 0.0001; H_2_O_2_ vs. control, *p* < 0.001; Fig. 4d). Even when differentiating between the anterograde and retrograde trajectories, the differences between these insults (LPS or H_2_O_2_) and the controls persisted (Fig. 4e). However, retrograde transport was affected distinctly and while inflammation severely decreased retrograde mitochondrial transport, it was enhanced by oxidative stress (Fig. 4b). In summary, neuroinflammation and oxidative stress severely affect the speed of axonal mitochondrial transport, whereby inflammatory damage preferentially impaired retrograde transport.

### Increased respiratory capacity in response to neuroinflammation and oxidative stress

Mitochondrial function in the cerebellar slice cultures was analyzed by using high-resolution respirometry. Since the cerebellar tissue is homogenized for these assays, the signal obtained comes from all the cell types in the tissue, including glial cells, and thus these results cannot be related directly to the observations in single axonal mitochondria described above. The two main processes related to mitochondrial respiration were analyzed: the transport of electrons obtained from NADH and FADH_2_ oxidation through the electron transport system (formed by complexes I to IV), which generates a proton gradient in the intermembrane space (through the respirometry assays and the analysis of different respiratory states; Table 2), and the oxidative phosphorylation, or ATP synthesis from ADP and inorganic phosphorus (Pi) catalyzed by ATP synthase (complex V), which uses the electrochemical gradient generated by electron transport as a driving force (measured as the ATP produced in the cultures).

**Table 2:** Respiratory states analyzed in the SUIT protocol designed for high-resolution respirometry assays

Respiratory state	Meaning
LEAK	Respiration state uncoupled from ATP synthase activity, due to proton leakage and the circuit of electrons initiated after the addition of malate and glutamate in the absence of ADP
Complex I (CI)	Respiration state of NADH-dependent complex I after the addition of ADP, in which the proton gradient of the intermembrane space is partially used by ATP synthase for oxidative phosphorylation (coupled respiration) and partially dissipated due to proton leakage (uncoupled respiration)
Complexes I and II (CI + CII)	Measurement of FADH_2_-dependent complex II respiration together with complex I respiration after the addition of succinate
Uncoupling	Endogenous respiration uncoupled from ATP synthesis through complex I and II substrates after the addition of oligomycin (ATP synthase inhibitor)
Electron transfer system capacity I + II (ETS I + II)	Measurement of the electron transfer system’s maximum capacity through the non-physiological uncoupling of the internal mitochondrial membrane after the addition of FCCP (an ionophore or uncoupling agent that dissipates H^+^)
Electron transfer system capacity II (ETS II)	Maximum respiration that only corresponds to complex II after the addition of rotenone (complex I inhibitor)
Complex IV (CIV)	Respiration state of complex IV after complex III inhibition by antimycin A, and the addition of ascorbate and TMPD

Explanation of all the mitochondrial respiratory states included in the SUIT protocol designed for high-resolution respirometry assays. The respiratory states were measured in a continuous manner (without breaks between enzymatic reactions), and in this way, the substrates from the first respiratory states are available for the following states

**Fig. 5:**
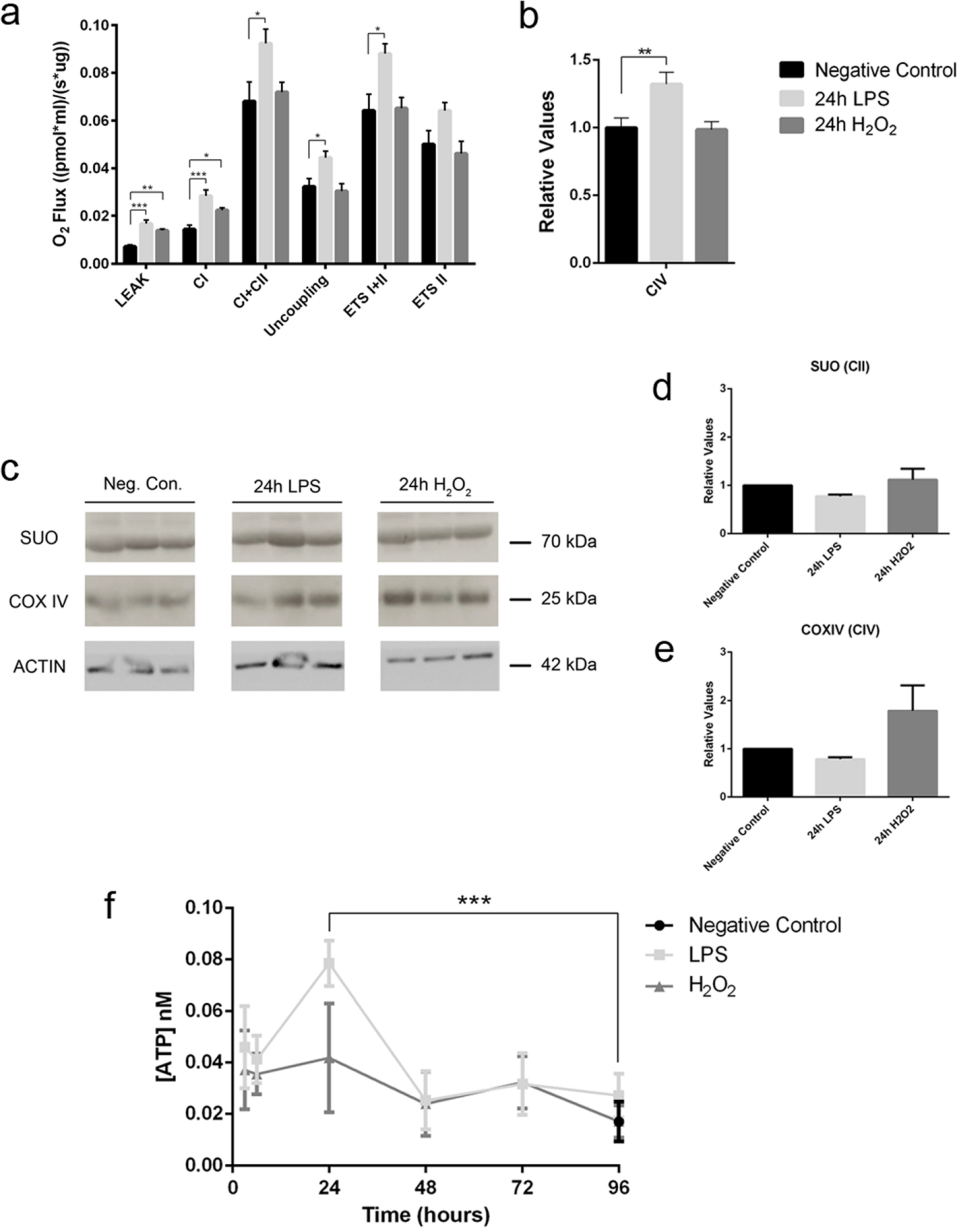
Effects of neuroinflammation and oxidative stress on mitochondrial function in cerebellar slice cultures. **a** Respiratory values of the cerebellar slice cultures show that neuroinflammation (LPS) produces an increase of LEAK, CI, CI + CII, uncoupling, and ETS I + II respiratory states, while oxidative stress (H_2_O_2_) causes an increase of LEAK and CI respiratory states (*n* = 13 controls, 9 LPS and 5 H_2_O_2_). One-way ANOVA and Dunnett’s multiple comparisons *post hoc* test for the respiratory states in which samples have a Gaussian distribution, Kruskal-Wallis test and Mann Whitney *U* test plus Bonferroni’s correction *post hoc* multiple comparisons for the respiratory states in which samples do not have a Gaussian distribution (mean ± SEM; *p < 0.05, **p < 0.01, ***p < 0.001). **b** Respiratory values for mitochondrial complex IV were obtained in different experiments for LPS and H_2_O_2_ stimulations. Therefore, CIV values were normalized with the respiratory values of the negative control in each experiment. The resulting relative values show that only LPS produces an increase of CIV respiratory state (controls *n* = 12, LPS *n* = 9; H_2_O_2_ *n* = 7). One-way ANOVA and Dunnett’s multiple comparisons *post hoc* test (mean ± SEM; **p < 0.01). **c** Western blot showing the expression of two subunits from two different mitochondrial electron transport chain complexes in the organotypic cerebellar slice cultures: the 70-kDa Fp subunit of the succinate ubiquinone oxidoreductase or complex II (SUO); and subunit IV of the cytochrome c oxidase or complex IV (COX IV). **d**, **e** Quantification of the Western blot shows no significant differences in SUO or COX IV expression (*n* = 3, one-way ANOVA; mean ± SEM). **f** The LPS challenge significantly augments ATP production in the organotypic cerebellar slice cultures at 24 h (*n* = 3, two-way ANOVA and Dunnett’s multiple comparisons post hoc test; mean ± SEM; ***p < 0.001)

The initial LEAK state of uncoupled respiration and NADH-dependent complex I respiration (CI) increased in conditions of neuroinflammation (LPS) compared to the control (*p* < 0.0001 for LEAK and *p* < 0.0001 for CI, one-way ANOVA and Dunnett’s multiple comparisons *post hoc* test; Fig. 5a). However, complex II was not affected, as the ETS II state (after the inhibition of complex I) was not different from the controls (*p* = 0.107). Therefore, the differences in the CI + CII (*p* = 0.048), uncoupling (*p* = 0.023), and ETS I + II (*p* = 0.018) states observed following LPS stimulation were mainly due to enhanced complex I respiration (Fig. 5a). During oxidative stress (H_2_O_2_), only the initial LEAK state of uncoupled respiration (*p* = 0.002) and NADH-dependent complex I respiration (CI) (*p* = 0.041) were enhanced relative to the controls (Fig. 5a).

The order in which complex IV substrates (ascorbate and TMPD) were added in the SUIT protocol was changed and optimized from H_2_O_2_ stimulations to LPS stimulations. Therefore, to be able to compare those measurements, LPS and H_2_O_2_ complex IV (CIV) respiratory values were normalized to their corresponding negative control respiratory values. Complex IV respiration increased after LPS stimulation (*p* = 0.009) but not after H_2_O_2_ stimulation (*p* = 0.990) (Fig. 5b).

To determine whether the increased respiration of some mitochondrial complexes was triggered by the increase of their enzymatic/respiratory capacity or by increased protein expression, two mitochondrial complexes were analyzed by Western blots (Fig. 5c): the succinate ubiquinone oxidoreductase or complex II (SUO) and the cytochrome c oxidase or complex IV (COX IV). The expression of SUO and COX IV did not change significantly following either challenge (SUO, *p* = 0.254 one-way ANOVA; COX, *p* = 0.118 one-way ANOVA; Fig. 5d, e). In summary, the acute response of the cerebellar tissue to either inflammation or oxidative stress involves an increase in respiratory capacity, mainly through complexes I and IV, although we cannot define which cell type was responsible for this process.

### Neuroinflammation induces a parallel increase in ATP production and respiratory activity in stressed cerebellar slice cultures

To determine whether the increase in the respiratory capacity of some mitochondrial complexes in the cerebellar slice cultures induced an increment in ATP production, we quantified ATP in a bioluminescent assay. Accordingly, ATP production appeared to increase after LPS stimulation (*p* = 0.0007), although this increase was transient and peaked at 24 h, it was no longer evident after 96 h (Fig. 5f). By contrast, no significant increase in the production of ATP was provoked by oxidative stress.

## Discussion

In this study, the acute effects of neuroinflammation and oxidative stress on axonal mitochondria have been analyzed, particularly with respect to their morphology and transport. Likewise, the functional consequences of these insults were assessed in terms of respiratory function and ATP production. We found that in response to either challenge, axonal mitochondria respond by increasing their size and cristae complexity, which may suggest an effort to increase energy production following acute damage. Mitochondria control their morphology through fusion and fission processes. Thus, the increase in axonal mitochondrial size suggests that the mitochondria might fuse to exchange mitochondrial components between damaged and healthy axonal mitochondria, thereby maintaining a functional population. Moreover, axonal mitochondria undergo an internal reorganization. The inner membrane structure is related to the mitochondrion’s metabolic state [28], and in more active respiratory states with high ATP concentrations, the mitochondria adopt a “condensed” morphology with a large number of cristae [27]. These results suggest that the mitochondria respond to the increased metabolic needs of axons during acute inflammation and oxidative stress.

The morphological alterations to the mitochondria observed during neuroinflammation occurred in parallel to the enhanced respiratory function of some mitochondrial complexes and the production of ATP by the tissue. Although we cannot determine which cell types were responsible for the increase in respiration and ATP, we predict glial cells will have a strong influence, such as microglia or astrocytes. However, the morphological changes in axonal mitochondria suggest that such changes contribute to the enhanced respiratory capacity and energy production soon after damage (within 24 h). We did not assess longer times because we were interested in early events, although several studies indicate that energy production decreases in the central nervous system (CNS) of animals suffering long-term inflammatory diseases such as EAE [35, 36].

Mitochondrial electron transport is the main biological process leading to ROS generation [37, 38], and higher metabolic rates correlate with higher levels of oxidative stress [39]. Indeed, the transient increase of ATP production (peaked after 24 h of LPS stimulation) correlates with an increased oxidative stress in the tissue confirmed by an increment of iNOS expression and ROS levels [20]. In that study [20], we quantified iNOS expression (by Western blot) and ROS production (using a fluorescent assay with H_2_DCFDA) at different times after LPS challenge (0–96 h). iNOS protein levels increased at 12–24 h after LPS challenge and decreased subsequently, and ROS levels peaked at 12 h and remained increased up to the study end point. Active mitochondria producing high levels of ATP in high metabolic states also produce more ROS, and probably those high ROS levels damage the mitochondria and impede the production of high ATP levels in longer time points rather than 24 h.

We also found that mitochondrial transportation is the earliest dysfunction of axonal mitochondria in response to neuroinflammation and oxidative stress. A strong decrease in the mean transport velocity was provoked by both insults and both directions, anterograde and retrograde. This is in accordance with the impaired axonal transport seen previously, even before axonal demyelination or structural alterations were observed [19]. This decrease in the mean velocity of axonal mitochondrial transport is critical because axons are very long and they have very active structures that require significant amounts of energy, such as synapses, nodes of Ranvier, active growth cones, or axonal branches [40, 41, 29]. If mitochondrial transport is impaired in axons, there would be a decrease in the energy (ATP) available at these sites of strong demand. In the nodes of Ranvier, such deficits would impair the activity of the Na^+^/K^+^ ATPase, which would in turn promote the reversion of axonal membrane Na^+^/Ca^2+^ transporters and provoke an increase in Ca^2+^ levels in the axoplasm that could trigger several degenerative processes [42, 16].

Interestingly, we found that each insult has a specific effect on the mitochondria. The mitochondria adopt a more rounded shape in response to oxidative stress, like the mitochondria of swollen axons in the EAE model [13]. Furthermore, while ATP production did not increase, the respiratory capacity of the LEAK state and CI increased less than during inflammation. Both observations indicate that oxidative stress is more directly damaging the mitochondria than inflammation. In terms of axonal mitochondrial transport, although transportation speed was severely reduced by both insults, neuroinflammation augments the number of stationary mitochondria and dampens retrograde mitochondrial motility, whereas oxidative stress increases the amount of retrograde motile mitochondria. The fact that retrograde mitochondrial transport increases after oxidative damage suggests that early damaged mitochondria are transported to the soma for elimination, yet additional mitochondria are not transported anterogradely because they may became not functional. By contrast, the increase in the number of stationary mitochondria during neuroinflammation is consistent with the increased density of stationary mitochondria in EAE axons seen previously [19]. Given that the size of the stationary sites is smaller following neuroinflammation than following oxidative stress, we propose that the mitochondria that were motile before the insult (motile mitochondria are smaller than stationary mitochondria [43]) stop and accumulate all along the axon during inflammation.

Regarding the differential mechanisms of axonal damage associated with CNS inflammation, we previously described that LPS triggers microglia activation in this ex vivo model of cerebellar slice cultures, which secrete pro-inflammatory cytokines and produce reactive oxygen species [20]. In this model, axonal damage seems to be highly dependent on oxidative stress, whereas pro-inflammatory cytokines contribute significantly to demyelination. The differential effects of such challenges in this study suggest that pro-inflammatory mediators have a significant effect on mitochondrial axonal transport, either through directly damaging axons or as an indirect effect of acute demyelination. Most importantly, at the time, we observed mitochondrial morphological changes and the disruption of axonal mitochondrial transport; axons were already damaged to some extent [20]. Both stimuli significantly increased the amount of dephosphorylated neurofilaments, indicative of the onset of axonal damage. Previous studies [2] have shown dephosphorylated neurofilaments being present in acute lesions from MS patients. Therefore, all the mitochondrial changes that we observed appear to occur at the initial stages of axonal damage either during neuroinflammation or oxidative stress.

It remains unclear what might cause the dramatic decrease in axonal mitochondrial transport velocity, either in neuroinflammation or oxidative stress. Considering that both anterograde and retrograde transport is affected, the different motor proteins in charge of such transport (kinesins for anterograde and dyneins for retrograde transport) may not be the primary cause. Therefore, we hypothesize that inflammatory and oxidative processes somehow alter the attachment of motor/adaptor/mitochondrial complexes to microtubules. Identifying the specific sites of damage in the axon cargo system will be critical to develop therapeutic strategies that prevent permanent axonal damage and disability.

**Fig. 6:**
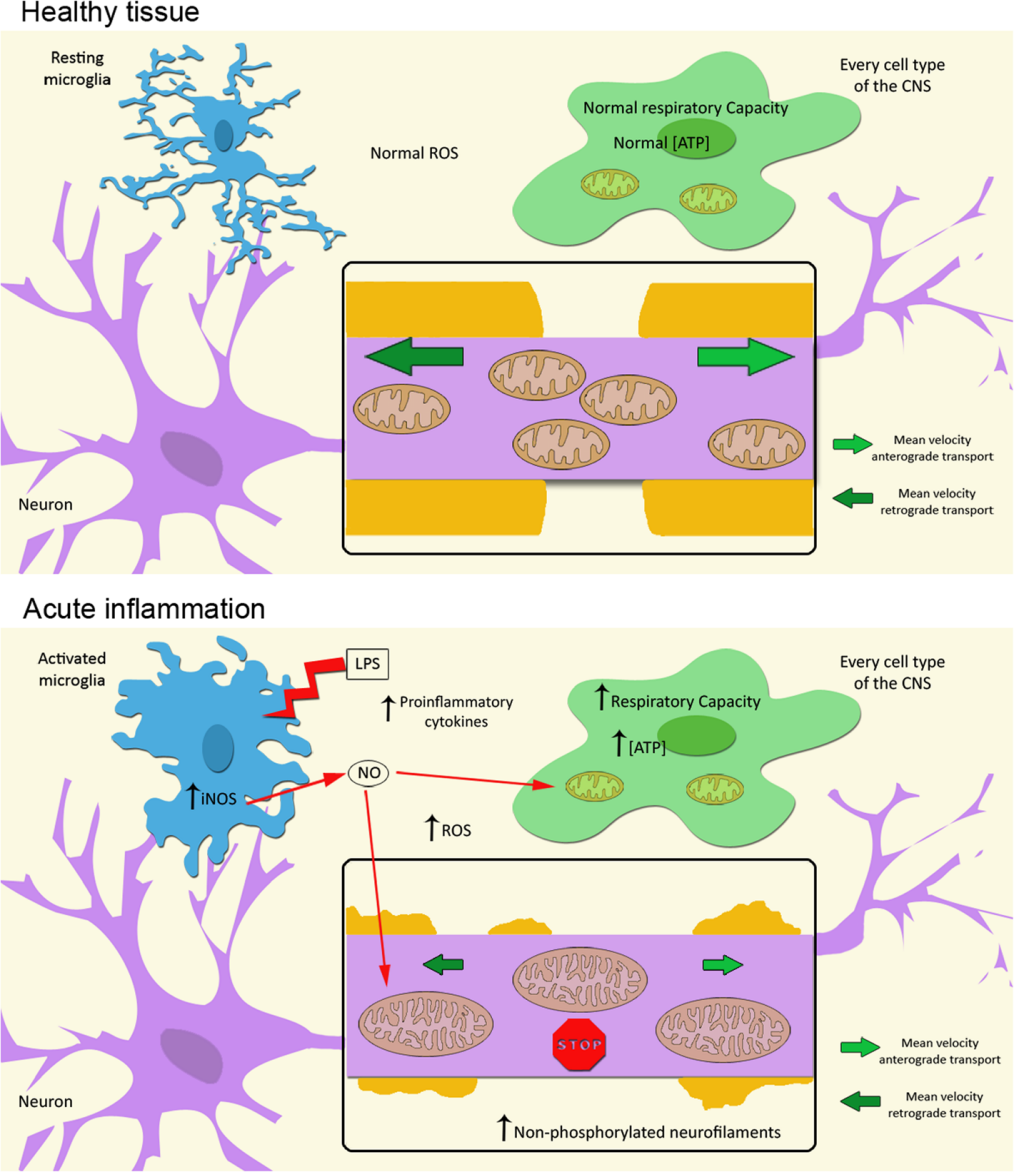
Mitochondrial response to acute neuroinflammation. The model of the mitochondrial response to an acute neuroinflammatory environment proposed shows that the mitochondria respond at the tissue level by increasing their respiratory capacity and ATP production, whereas at the axonal level mitochondria respond by increasing their size and cristae complexity, although their transport is disrupted. Mitochondrial paralysis occurs early, before axonal damage is irreversible. Therefore, disruption of axonal mitochondrial transport (mainly retrograde) is a critical mechanism underlying mitochondrial dysfunction during neuroinflammation. Our results, and previous studies in this model [20], suggest that preserving axonal mitochondrial transport could represent a neuroprotective therapy for acute CNS inflammation

Our study focused on the acute response to inflammatory and oxidative stress in order to identify the earliest process involved in axonal degeneration. Although this is important to identify future neuroprotective strategies, our results cannot explain how the CNS responds to chronic damage. Our data was obtained in an ex vivo model in order to benefit from high-resolution imaging techniques, and they are in agreement with previous observations made in the EAE animal model [19]. We found that the main complexes that react to neuroinflammation, complexes I and IV, are the same as those preferentially altered in brain lesions of MS patients [17, 35, 44]. Therefore, and based on current evidence, we hypothesize that after acute inflammation such as MS relapses, the mitochondria compensate for the extra energy demands by increasing their size, cristae complexity, and the respiratory capacity of complexes I and IV. However, due to impairment in axonal mitochondrial transport, axons will suffer energy delivery depletion at sites of high-energy demand such as the nodes of Ranvier. This energy delivery depletion, in addition to other factors triggered by inflammation, will promote axonal damage (Fig. 6).

## Conclusions

The results presented here show that after acute inflammation and oxidative stress in the CNS, axonal mitochondria respond to the augmented energy demands by increasing their size and cristae complexity. However, axonal mitochondrial transport is rapidly and severely impaired, preventing the delivery of energy along axons and to specific sites of high-energy demands. Such unattended energy demands may trigger several degenerative processes, leading to axonal damage and subsequent transection. Therefore, addressing these axonal mitochondrial transport deficits could revert the progression of axonal damage to irreversible axonal degeneration.

## Abbreviations

ADP: adenosin diphosphate
ANOVA: analysis of variance
AR: aspect ratio
ATP: adenosin triphosphate
CI: complex I
CII: complex II
CIV: complex IV
CNS: central nervous system
COX IV: sytochrome c oxidase subunit IV
DIV: days in vitro
EAE: experimental autoimmune encephalomyelitis
ETS: electron transfer system
FADH_2_: reduced flavin adenine dinucleotide
FCCP: carbonyl cyanide-*p*-trifluoromethoxyphenylhydrazone
H_2_O_2_: hydrogen peroxide
LEAK: leak oxygen flux
LPS: lipopolysaccharide
MS: multiple sclerosis
NADH: reduced nicotinamide adenine dinucleotide
OXPHOS: oxidative phosphorylation
ROX: residual oxygen consumption
SUIT: substrate-uncoupler-inhibitor-titration
SUO: succinate ubiquinone oxidoreductase
TEM: transmission electron microscopy
TMPD: *N*,*N*,*N*′,*N*′-tetramethyl-*p*-phenylenediamine dihydrochloride
