# Correction to: the disruption of mitochondrial axonal transport is an early event in neuroinflammation

**DOI:** 10.1186/s12974-017-0979-2

**Published:** 2017-10-23

**Authors:** Oihana Errea, Beatriz Moreno, Alba Gonzalez-Franquesa, Pablo M. Garcia-Roves, Pablo Villoslada

**Affiliations:** 1 Center of Neuroimmunology, Institut d’Investigacions Biomèdiques August Pi Sunyer (IDIBAPS), Cellex Building, Laboratory 3A, Casanova 145, 08036 Barcelona, Spain; 2 Diabetes and Obesity Research Laboratory, Institut d’Investigacions Biomèdiques August Pi Sunyer (IDIBAPS), 08036 Barcelona, Spain; 3 Spanish Biomedical Research Center in Diabetes and associated disorders (CIBERDEM), University of Barcelona, 08907 Barcelona, Spain; 4 Department of Physiological Sciences II, University of Barcelona, 08907 Barcelona, Spain; 5 University of California, San Francisco, USA

## Correction

After publication of the article [1], it has been brought to our attention that the full funding acknowledgement is missing from the original article. It should also include the following –

“This work was supported by the Instituto de Salud Carlos III with FEDER funds (Otra forma de hacer Europa) from the European Commission (FIS: PI12/01823)”.
